# Impact of Transmyocardial laser revascularization on Pathomorphological and Physiological Patterns of Myocardial Microcirculation in patients with advanced CAD

**DOI:** 10.1186/1749-8090-10-S1-A88

**Published:** 2015-12-16

**Authors:** Ilya Berishvili, Tamara Artuhina, Marat Semenov

**Affiliations:** 1 TMR Department, Bakoulev Cardiovascular Scientific Center, Moscow, 199991, Russian Federation

## Background/Introduction

The mechanism by which transmyocardial revascularization (TMR) offers clinical benefit is controversial. We hypothesized that TMR ameliorates vasoconstriction in patients with ENDCAD

## Aims/Objectives

This study was undertaken to demonstrate that transmyocardial laser revascularization in patients with advanced CAD improves results of combined operations CABG+ TMR through reduction of vasoconstriction

## Method

We investigated vessels of coronary microvascular network of patients with ENDCAD and analyzed the histological changes in two groups: in group of patients died after sole CABG (1-st group - 8 heart specimens) and group of patients died after combined operations CABG+TMR (2-nd group - 6 heart specimens).These data are compared with hospital results (deaths, MACE) in two large groups of patients with ENDCAD: with isolated CABG (1-st group -33 operations) and CABG+TMR (2-nd group - 87 operations), operated in 2011-2012.

## Results

in all hearts after sole CABG was identified coronary arteriolar vasospasm that decreases coronary and bypass flow and thus increase the probability of thrombi formation. All cases after CABG+TMR revealed vasodilatation in lased areas. Reduction in the occurrence of vasospasm in cases with CABG+TMR can prevent graft and coronary occlusion. High indices of hospital mortality (12.1%) and morbidity(33.3%) in cases with sole CABG can be explained with coronary spasm in patients with advanced CAD. On the other hand, reduced hospital mortality (1.15%) and morbidity (2.3%) in the second group(CABG+TMR) can be explained by laser-influenced vasodilatation of distal coronary bed. Elevated resistance of the coronary bed hinders the effectiveness of the graft and myocardial blood flow(MBF). We suggest, that intraoperative effectiveness of TMR based of denervation, dilatation of microvascular network of the myocardium and intraoperative improvement of perfusion.

## Discussion/Conclusion

elevated resistance of myocardial microcirculation(vasoconstriction) in patients with advanced CAD inhibit the effectiveness of the CABG. In cases with TMR effectiveness is most likely due to vasodilatation of vasoconstriction of myocardial microcirculation

